# Impact of accelerated review policy on portfolio planning of vaccine companies

**DOI:** 10.3389/fpubh.2024.1339141

**Published:** 2024-12-09

**Authors:** Guicui Liu, Zhe Huang

**Affiliations:** ^1^School of Business Administration, Shenyang Pharmaceutical University, Shenyang, China; ^2^Drug Regulatory Research Base of NMPA - Research Institute of Drug Regulatory Science, Shenyang Pharmaceutical University, Shenyang, China

**Keywords:** mixed-integer linear programming, accelerated review, capacity planning, vaccine companies, optimization

## Abstract

**Background:**

With the introduction of the accelerated drug review policy in China, the clinical research and development time and the review and approval time of drugs have been shortened accordingly. Especially under the influence of the COVID-19 pandemic, the vaccine formulations released through the accelerated review policy are springing up, and the question of how the accelerated review policy affects the investment portfolio of vaccine enterprises has also attracted more and more attention.

**Aims and methods:**

The article uses mixed-integer linear programming to develop a new model on portfolio planning for vaccine companies based on the accelerated review policy context. The model is constructed using the Gurobi extension class of .NET, and the investment decision is made and simulated by the Gurobi solver to investigate the portfolio planning decision of a vaccine company maximizing the net present value of its vaccine production portfolio with the increase of available capital over a 20-year time horizon.

**Results:**

The NPV under the accelerated review policy is significantly higher than the net present value under the standard review policy when the available capital exceeds RMB 900 million. And the difference between the two of them peaks at RMB 1.87 billion when the available capital is RMB 1.9 billion; break-even occurs about 1.3 years earlier in the accelerated review policy than in the standard review; and when the available capital is the same, firms in the accelerated review policy choose to produce four products earlier and make the decision to invest in facility construction earlier; scenarios in the accelerated review policy are not as sensitive to changes in model parameters as they are in the standard review.

**Conclusion:**

The accelerated review policy is effective in providing incentives for commercialisation. The results of this study will provide an effective reference for vaccine companies to make scientific portfolio planning under the accelerated review policy.

## 1 Introduction

Being able to quickly obtain innovative drugs with obvious clinical advantages has always been an urgent need for millions of patients. Traditional access to new drugs is often time-consuming and labor-intensive, and products need to go through a long ice-breaking period to open up the market, while the policy of accelerating the review and approval of drugs on the market opens up a fast-track for the entry of innovative drugs into the market, benefiting thousands of patients in urgent need of medication. Good regulation is better able to maintain market dynamics and encourage innovation while protecting public health (1). The United States began to pay attention to procedures related to accelerating the review of drug registrations at an early stage and established priority review and priority review in 1992, after which other pathways for accelerated review were added one after another (2). Japan has also introduced three special review models for new drugs (3). China’s pharmaceutical industry has a huge output value, but the pharmaceutical market is not standardized enough and there are not enough innovative medicines. In order to better meet the international pharmaceutical market and effectively solve the problem of drug review backlog, China attaches great importance to the drug review work, vigorously encourages the research and development of new medicines, and includes accelerating the drug review work as an important part of the “14th Five-Year Plan” (4). The newly revised Measures for the Administration of Drug Registration (5) clearly put forward four accelerated review policy, namely special approval, priority review, breakthrough therapy designation, and conditional approval. And in the context of the new pharmaceutical policy, how to seize the policy dividend, based on the market capacity, to better develop a high-quality portfolio planning decision has become an urgent problem for vaccine companies.

At present, China’s research on the accelerated review policy mainly stays on the research on the content of the accelerated drug review policy in Europe, the United States, Japan and China (6–9), as well as the impact of a single accelerated review policy on the time to market, quantity and quality of medicines (10–15), while there are fewer studies on the extent of the impact of the joint use of the four accelerated review channels of new drugs in China on the time of development and approval of new drugs. To fill this gap, this study will select 28 new drugs with registration type I and make an in-depth study of their clinical development time and marketing review time, which will be discussed in the third part of the article.

In addition, the unnecessary investment costs brought by ineffective capacity planning and investment planning will not only make the enterprise resources wasteful by excessive attrition, but will even make the enterprise face an existential crisis. Currently, most scholars construct mixed integer linear programming (MILP) models based on different stages of the drug life cycle or several adjacent stages to optimize the drug candidate portfolio, licensing deal portfolio, facility and equipment investment portfolio, etc., so as to provide guidance for the future development planning of enterprises (16–21). However, existing models do not explore firms’ portfolio planning (including timing of capital investment and portfolio choice, etc.) based on the accelerated review policy, nor do they explore the impact of the accelerated review policy on firms’ future investment strategies. Knowing exactly how the combined use of the four accelerated review channels will benefit a company’s future strategic planning, it is essential to incorporate the accelerated review policy into the portfolio planning model.

Based on the above considerations, the contributions made in this article, based on existing literature, are as follows: considering the background of the accelerated review policy, based on the premise of the joint use of multiple accelerated review channels, constructing the MILP mathematical model, and exploring the enterprise’s portfolio planning decision-making with the goal of maximizing the net present value of the vaccine production portfolio; and the simulation analysis further dissects the facility and equipment investment planning and product mix planning of enterprises in different policy contexts, and by virtue of it, proves the applicability and validity of the model.

## 2 Literature reviews

This section will review the following two categories of relevant literature.

### 2.1 Accelerated review of regulatory designations

With the introduction of these four channels in China and the improvement of the subsequent supporting mechanisms, a large number of clinically urgent drugs have entered the review of the “fast track,” the clinical development time and review and approval time of many new drugs have been shortened accordingly, and a number of new drugs with obvious clinical value to meet the urgent needs of new drugs have been approved and marketed in advance (22). Su et al. (23) studied the development trend of innovative drugs in China in recent years, and 69% of the innovative drugs received priority review, the accelerated review policy may become a required channel for drug companies to quickly bring new drugs into the market. In order to accelerate the effective implementation of China’s accelerated review policy, scholars have made fine studies on different channels. Feng et al. (24) and Gao et al. (25) made an in-depth study on the priority drug review channel, while Hu et al. (26) and Zhao et al. (8) studied the breakthrough therapy and conditional approval channels, pointed out the existing problems, and put forward rationalization proposals; different scholars also discussed the factors affecting the review of new medicines; different scholars have also discussed the factors affecting the review of new drugs, and they have mainly explored in detail the four aspects of the system itself, the policy implementation process, the review concept and the impact on the industry (27–30). Similarly, a comparative analysis of the system, mechanism and implementation performance of the special review model for new drugs in the United States, the European Union and Japan, as well as drawing on their implementation experience, will be conducive to the gradual establishment and improvement of a multi-channel special review model system for new drugs in China (6). In addition, there are some scholars who reveal the role of accelerated review policy on the review timeframe of new drugs through the review and approval data of new drug launches, Ren et al. (12) based on the new drug launches and approvals in China, the United States, and the European Union from 2009 to 2018 to derive the trend of change in the median value of the review timeframe of new drugs in China, the United States, and the European Union under the designation of the fast-track channel. However, it is still unclear to what extent the combined use of these channels influences the time spent on regulatory review process. Therefore, this paper will further consider to what extent the joint use of the four special review modes for new drugs in China affects the time taken to develop and approve new drugs after the introduction of the newly revised Administrative Measures for Drug Registration.

### 2.2 Vaccine enterprise investment portfolio planning

Vaccines are unique medicines that pose challenges for their manufacture and marketing due to the demanding nature of the basic raw materials, the microorganisms themselves, the storage and transportation conditions, the complexity of the manufacturing process (31), and the high level of industry regulation. And these challenges will have implications for the development of commercialization strategies for vaccine companies. Applying mixed integer linear programming (MILP) models to the R&D, production and sales of vaccine companies can provide more support and reference for future strategic decisions. Rogers et al. (17) proposed a stochastic optimisation model using MILP to construct drug R&D portfolio management early on to select optimal product portfolios among different stages of drug candidates, different levels of market and technological uncertainty. Many scholars have since conducted extensive research on the different stages of the life cycle. Gökalp et al. (19) explored a model of approximate dynamic portfolio optimisation for the clinical phase of a drug product, in order to be able to find the better product portfolio solution faster. Plotkin et al. (32) proposed a portfolio model on the production phase, considering the complexity and cost drivers associated with vaccine production, with a view to informing business decisions. Maranas et al. (18) focused on exploring portfolio models of pharmaceutical companies in the early days regarding the period of licensing agreements and R&D investments. There are also scholars who focus on the logistics and distribution chain of vaccines, such as Manupati et al. (33) by formulating a mixed integer linear programming (MILP) model for locating and allocating cold storage facilities for batch vaccine production. Different influencing factors are considered in different segments, e.g., Hesarsorkh et al. (21), Tsang et al. (34) proposed a new mixed integer linear programming model for optimal R&D portfolio selection taking into account the details of processing time, investment cost, and production scale. Papageorgiou et al. (20), on the other hand, explored capacity management of product portfolios based on R&D costs, demand forecasting, production costs and resource allocation. However, these existing models do not consider the impact of the accelerated review policy on the strategic decisions of vaccine firms, such as the timing and portfolio of capital investments. Therefore, this article will develop a MILP model to compare the impact of accelerated review policy and standard review policies on the portfolio planning of vaccine firms and provide a reference for vaccine companies to make scientific commercialization strategies in different policy contexts.

## 3 Background of the model policy

Considering the impact of the accelerated review policy on the investment and production planning of vaccine enterprises, this paper will make a study on the clinical development time and marketing review time of 28 new drugs with the first marketing application since the implementation of the accelerated review policy in China until December 2022 and the registration type of Class I. Comparing the time spent on these two stages by new drugs marketed in the accelerated review policy and new drugs marketed through the standard review mode. The sources of data were the database of the Drug Evaluation Center of the State Drug Administration and the database of the Drug Fusion Cloud, and the 15th day of each month was used if there was no specific day (Table 1).

**Table 1:** New drug review list.

Drug name	First-time market application	First-in-class	Registration type	Review mode	NDA submitted date	NDA approval date	Development timeline (days)
Telitacicept for injection	yes	yes	1	Priority review, Special approval, Conditional approval	2019.11.13	2021.3.9	3,422
Pamiparib capsules	yes	yes	1	Priority review, Special approval, Conditional approval	2020.7.20	2021.4.30	1,720
Emelenamine tenofovir tablets	yes	yes	1	Priority review, Special approval	2020.9.18	2021.6.22	2,268
Herombopag olamine tablets	yes	yes	1	Priority review, Special approval, Conditional approval	2020.6.18	2021.6.16	3,333
Emitasvir phosphate capsules	yes	yes	1	Priority review, Special approval	2019.9.13	2020.12.21	2,053
Ravidasvir hydrochloride tablets	yes	yes	1	Priority review, Special approval	2018.8.6	2020.7.29	1,194
Edaravone dexitol injection	yes	yes	1	Priority review, Special approval	2018.11.1	2020.7.29	3,650
KW-136 capsule	yes	yes	1	Priority review, Special approval	2018.6.22	2020.2.11	1,265
Cyclopool emulsion injection	yes	yes	1	Priority review	2020.1.22	2021.2.2	—
Azvudine tablets	yes	yes	1	Priority review, Conditional approval	2020.7.9	2021.7.20	2,711
Contezolid tablets	yes	yes	1	Priority review, Special approval	2020.1.4	2021.6.1	3,624
Furmonertinib mesilate tablets	yes	yes	1	Priority review, Conditional approval	2019.12.10	2021.3.2	1,415
Hybutimibe tablets	yes	yes	1	Priority review	2019.1.17	2021.6.25	2,472
Savolitinib tablets	yes	yes	1	Priority review, Conditional approval	2020.6.6	2021.6.22	3,019
Candonilimab injection	yes	yes	1	Priority review, Conditional approval, breakthrough therapy designation	2021.9.26	2022.6.28	1,497
Disitamab vedotin for iicction	yes	yes	1	Priority review, Conditional approval, breakthrough therapy designation	2020.8.28	2021.6.8	2,455
Serplulimab injection	yes	yes	1	Priority review, Conditional approval	2021.4.23	2022.3.22	883
Icaritin	yes	yes	1.2	Priority review, Special approval, Conditional approval	2021.4.10	2022.1.10	4,541
Henagliflozin proline	yes	yes	1	Special approval	2020.9.30	2021.12.31	3,085
Enzalutamide injection	yes	yes	1	Priority review, Special approval, Priority review.	2020.12.21	2021.11.24	1,693
Baricitinib	yes	yes	1	Priority review, Conditional approval, breakthrough therapy designation	2020.10.10	2021.11.24	1,975
Ainuovirine (ACC007) tablets	yes	yes	1	Priority review, Special approval	2020.7.25	2021.6.25	1,354
ZL-2401 totoate tablets	yes	yes	1	Priority review, Special approval	2020.2.14	2021.12.14	693
Levornidazole disodium phosphate for injection	yes	yes	1	Priority review	2019.8.15	2021.5.26	3,948
SHR6390 Tablets	yes	yes	1	Priority review, Special approval, breakthrough therapy designation	2021.4.27	2022.1.5	2,507
Donafenib	yes	yes	1	Priority review, Special approval	2020.5.15	2021.6.8	3,061
Zimberelimab injection	yes	yes	1	Conditional approval	2020.2.21	2021.8.25	1,009

Based on Table 1 to calculate the median time for clinical development and median time for marketing review under different review policies, it is clearly concluded that the accelerated review policy can effectively shorten the time for clinical development and time for marketing review and approval of new drugs (Table 2).

**Table 2:** Comparison of clinical development time and market review time for different review modes.

	Clinical development time	Market review time
Accelerated review mode	5.62 years	1.06 years
Standard review mode	7.43 years	1.84 years (8)

## 4 Model

### 4.1 Problem statement

Considering the accelerated review policy, a strategic decision model on portfolio planning for vaccine firms is constructed to address the challenges faced by vaccine formulation firms in business development with cost and constraint equations divided into facility and equipment level, product level, and capital level, with the ultimate overarching goal of maximizing the NPV over a hypothetical time horizon.

The time span of this study is 20 years, reflecting the typical period of patent exclusivity prior to generic entry (35), and the time span is discretized into yearly time intervals. The production volume of products and the investment in facility equipment are subject to the limitation of available funds. This paper will establish a Mixed-Integer Linear Programming (MILP) model based on various factors such as annual available funds, market demand, and other considerations under different policy backgrounds. The model will be solved and optimized using the Gurobi solver in .NET. The available funds in the model range from 500 million RMB to 2.2 billion RMB. Based on the investigation, the given relevant information is as follows.

- The combination and planning time range of potential products.
- The level of demand for disease prevention and control institutions.
- The predicted selling price for each product.
- The production cost for each product.
- The facility size (small-scale, large-scale).
- The facility construction period and investment cost.
- The fixed operating cost of the facility.
- Interest rate.

Modeling based on the above information explores the following questions.

- How does the net present value (NPV) change with the change of available funds under the two policies?
- What is the optimal investment capital for enterprises under different policies?
- What kind of product portfolio decisions will companies make under different policies?
- What kind of facility investment plans will companies make under different policies?

### 4.2 Mathematical formulation

#### 4.2.1 Mathematical modeling of vaccine facilities and equipment

It takes a long time to build a facility based on the required process, and the time to completion is related to the size of the facility and the complexity of the process that the facility can provide for production. The time to build a completed facility is *τf_Fs_*. *A_Fs,t_* represents the number of facilities of size *Fs* available for production at year t. *Fac_Fs,t-τfFs_* denotes the amount of investment in the decision-making facility at year *t*. Under the condition that a firm makes an investment decision at moment *t*, the facility can produce vaccines only after it is fully established (Equation 1), i.e., in the model, there is no production until the construction of the facility is complete.


(1) $$AF_{s,t}\leq AF_{s,t-1}+Fac_{Fs,t-\tau f_{Fs}}$$

The investment cost of new facilities and equipment is *Inv_Fs_*, which includes the cost of construction and installation of modules, equipment procurement, engineering costs, basic process development and validation, but excludes the cost of land. Specific facilities and equipment include bioreactors, VPM filling machines, light plants, warehouses, QA/QC labs, office buildings, etc. If the decision to build certain facilities is made only in year t (i.e., the investment decision is set to 1), the cost of facility investment at year t is (Equation 2).


(2) $$Flt=\underset{Fs}{\sum }Fac_{Fs,t}*\mathrm{In}V_{Fs}$$

*Fixopt_Fs,t_* denotes the fixed operating cost (Equation 3) of an available facility of size *Fs*, including the fixed operating costs of heating, ventilation, and air-conditioning operation of the facility, and the operating cost is related to the size of the facility. The operating costs here are assumed to be about one-tenth of the investment cost of the facility, as analyzed by the relevant data (34). *TFixopt_t_* denotes the total operating cost of all available facilities at year *t* (Equation 4).


(3) $$\mathrm{Fixop}t_{Fs,t}=0.1*\mathrm{In}V_{Fs}$$

(4) $$\mathrm{TFixop}t_{t}=\underset{t}{\sum }\underset{Fs}{\sum }\mathrm{Fixop}t_{Fs,t}$$

#### 4.2.2 Mathematical modeling of vaccine products

Assuming that there are unique production lines for several products and common facilities and equipment in the production facility under the premise of meeting GMP cleanliness and risk criteria for the plant, and that the total quantity of certain products P produced in a facility is *TrtF_P,Fs,t_*, the capacity constraints for the facility can be expressed as follows: the sum of the quantities of the different types of products P produced in a given available facility cannot exceed the facility’s equipment’s annual maximum production capacity *M_Fs_* (Equation 5).


(5) $$\underset{P}{\sum }TrtF_{P,Fs,t}\leq M_{Fs}$$

As a characteristic of vaccines, all products produced by an enterprise must be used at the CDC institution or the vaccination unit designated by the CDC institution that initially demanded them (hereinafter, the default is to deliver the vaccines to the CDC institution), i.e., the vaccine P produced needs to supply the demand of the said institution in full; therefore, the total number of a vaccine product supplied by an enterprise to the CDC institution should be equal to the total number of that vaccine product produced at all facilities (Equations 4–6), i.e., by default, all marketable vaccines produced by the enterprise will be fully supplied to the market demand; at the same time, the total amount of vaccine P produced in all facilities should be less than or equal to the total demand for product P in the disease prevention and control organization (Equation 7). Where *TTP_p,t_* is the total amount of product P produced by all facilities in year *t*, and *Dd_P,t_* is the annual market demand for a vaccine product P in year *t*.


(6) $$TTP_{P,t}=TTD_{P,t}=\underset{Fs}{\sum }\sum TrtF_{P,Fs,t}$$

(7) $$TTP_{P,t}\leq Dd_{P,t}$$

The variable cost of product P*, VCCOG_p_* is the total basic cost of producing a product P, including a range of costs such as raw materials, consumables, and labor costs. Although scale production is a more common approach to vaccine production, better use of human resources and equipment can achieve some degree of scale cost savings. *TCOG_t_* is the total cost of production for all products in year t, which is calculated as shown in Equation 8.


(8) $$TCOG_{t}=\sum \mathrm{PVCCO}G_{P,Fs}$$

#### 4.2.3 Mathematical modeling of transportation

*Transport_t_* denotes the sum of transportation costs incurred throughout the year *t*, determined by the total number of doses (*Dd_t_*) shipped from the place of vaccine production to each disease prevention and control facility throughout the year *t* (Equation 9). Where *a* is the average transportation cost per dose of vaccine, the study assumes that transportation costs are consistent across vaccine product P.


(9) $$\mathrm{Transpor}t_{t}=a*Dd_{t}$$

#### 4.2.4 Mathematical modeling of income

The annual sales of a vaccine product P at year *t* (*SalesP,_t_*) are derived by multiplying the total number of doses of the vaccine product P sold at year *t* by the unit sales price of the product (Equation 10), the product can only be sold when it is authorized to be marketed, i.e., the product has a selling price after it has been authorized to be marketed; before that, depending on the actual situation, the model needs to be set up so that its sales price is equal to zero. Where *Price_P,t_* is the sales price of a product in year *t* (unit: 10,000 yuan).


(10) $$\mathrm{Sale}s_{P,Mkt}=\underset{Fs}{\sum }TTD_{P,t}*\mathrm{Pric}e_{P,t}\forall t\in Mkt$$

The manufacturer’s total revenue from vaccine sales in year *t* is calculated from the total sales of all developed and market-authorized vaccine products P in that year (Equation 11).


(11) $$TRev_{t}=\underset{P}{\sum }\mathrm{Salse}s_{P,t}$$

#### 4.2.5 Objective function and capital constraints

At the development stage, the total amount of available capital cannot exceed the total amount of investment capital raised by the firm. Therefore, the cash flow of the firm at each stage is always limited by the capital available at that moment, i.e., the cash flow at each stage cannot exceed the total amount of capital available at that point in time (Equation 12), and it is important to note that the capital available always needs to be greater than zero.


(12) $$\emptyset \leq \underset{t}{\sum }\mathrm{cflow}\;\forall \;t\in Mkt$$

The discount factor is the rate at which a unit of currency is converted to its present value at various future periods under conditions where interest is calculated on a compound interest basis. The discount factor is used to convert a future investment or cash flow to its present value using an interest rate (*r*), which is usually a trade-off factor used to reflect the present value of future cash flows (Equation 13).


(13) $$\delta_{t}=1/{\left(1+r\right)}^{t}$$

Over a period of time, it is assumed that a company’s objective is to maximize its return on capital by optimizing its capacity investment strategy through planning based on a portfolio of investments in different facilities and a portfolio of product development and production, e.g., by prioritizing products that will generate the greatest return on capital while minimizing capital expenditures. Cash flow for a given year is derived from the previous year’s cash flow plus the total revenue for that year minus the costs incurred in that year for production, labor, equipment, etc., and the cost of vaccine formulation wastage (Equation 15), which, according to China’s vaccine management regulations, should be within 5% (Equation 14). Cash flow (*cflow_t_*) is the total revenue (*TRev_t_*) minus the investment cost of facilities and equipment (*FI_t_*), fixed operating cost (*TFixopt_t_*), total variable cost of products (*TCOG_t_*), transportation cost (*Transport_t_*), and vaccine wastage cost (*Wastaget*) (Equation 15).


(14) $$\mathrm{Wastag}e_{t}=5\%*\underset{P}{\sum }TTP_{P,t}*\mathrm{Pric}e_{P,t}$$

(15) $$\begin{array}{l} \mathrm{cflo}w_{t}=TRev_{t}-Fl_{t}-\mathrm{TFixop}t_{t}\\ \;-TCOG_{t}-\mathrm{Transpor}t_{t}-\mathrm{Wastag}e_{t} \end{array}$$

In this study, the overall objective of this MILP model is to maximize the NPV by comparing different portfolios. The expected NPV over a certain time horizon is obtained by calculating the sum of discounted cash flows for each year (Equation 16). The impact of the accelerated review policy on firm-related decisions is also explored by comparing the results of the calculations under different policy conditions.


(16) $$NPV=\underset{t}{\sum }\mathrm{cflo}w_{t}*\delta_{t}$$

## 5 Algorithm

**Figure 1:**
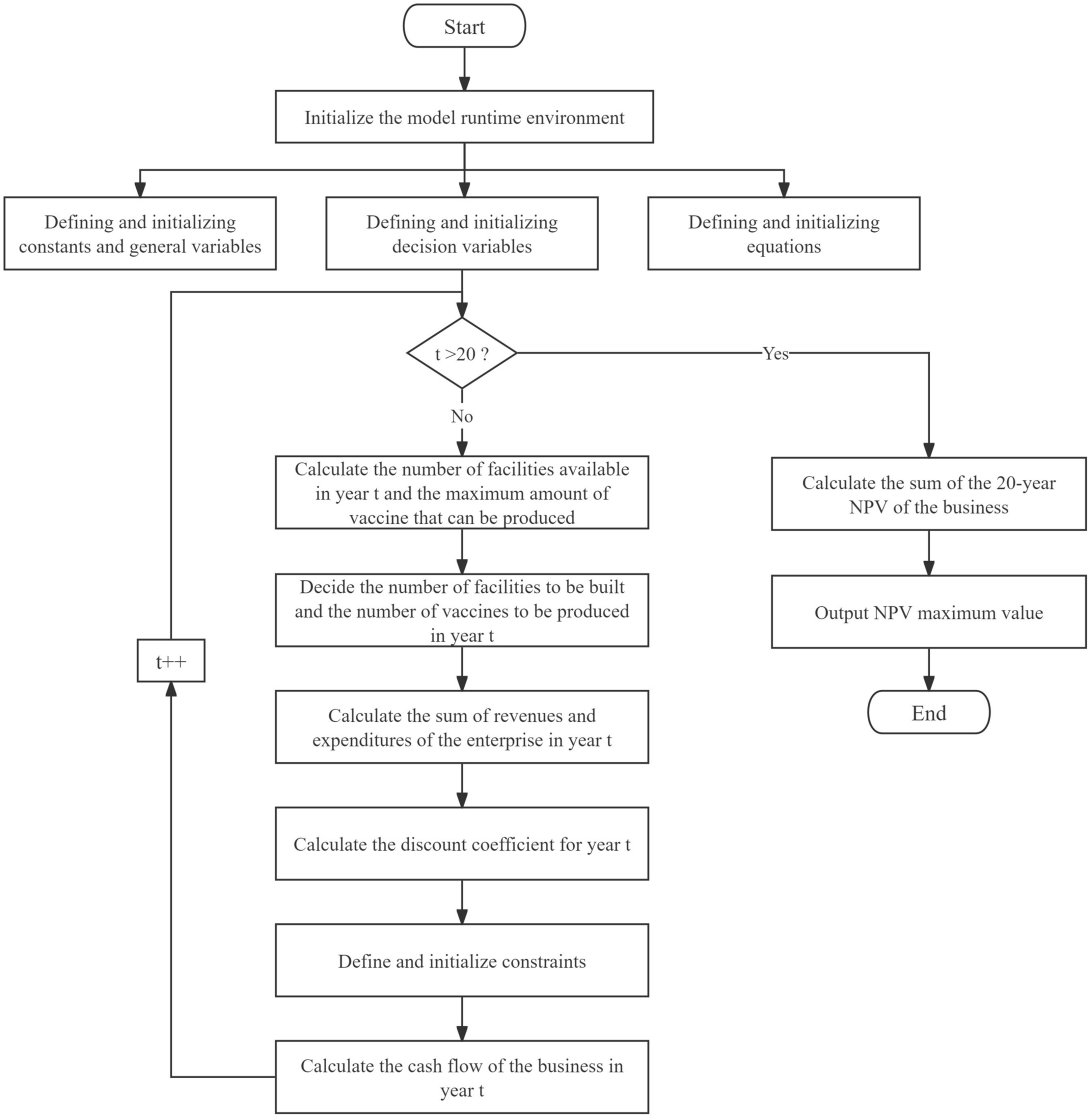
Algorithm flow chart.

The model used in the study is a mixed integer linear programming model and the model in the paper is modeled through .NET and solved optimally using the Gurobi solver under .NET. The algorithmic flow of the model is shown in Figure 1.

## 6 Computational experiments

### 6.1 Case description and data

In this article, data simulation and program construction are conducted based on four representative vaccine products. The data is collected and analyzed from annual reports of the company, the National Institutes for Food and Drug Control, China Association for Pharmaceutical Research and Development, Intelligence Research Database, China Commercial Intelligence Network, World Health Organization (WHO), and consultations with experts.

#### 6.1.1 Product data

**Figure 2:**
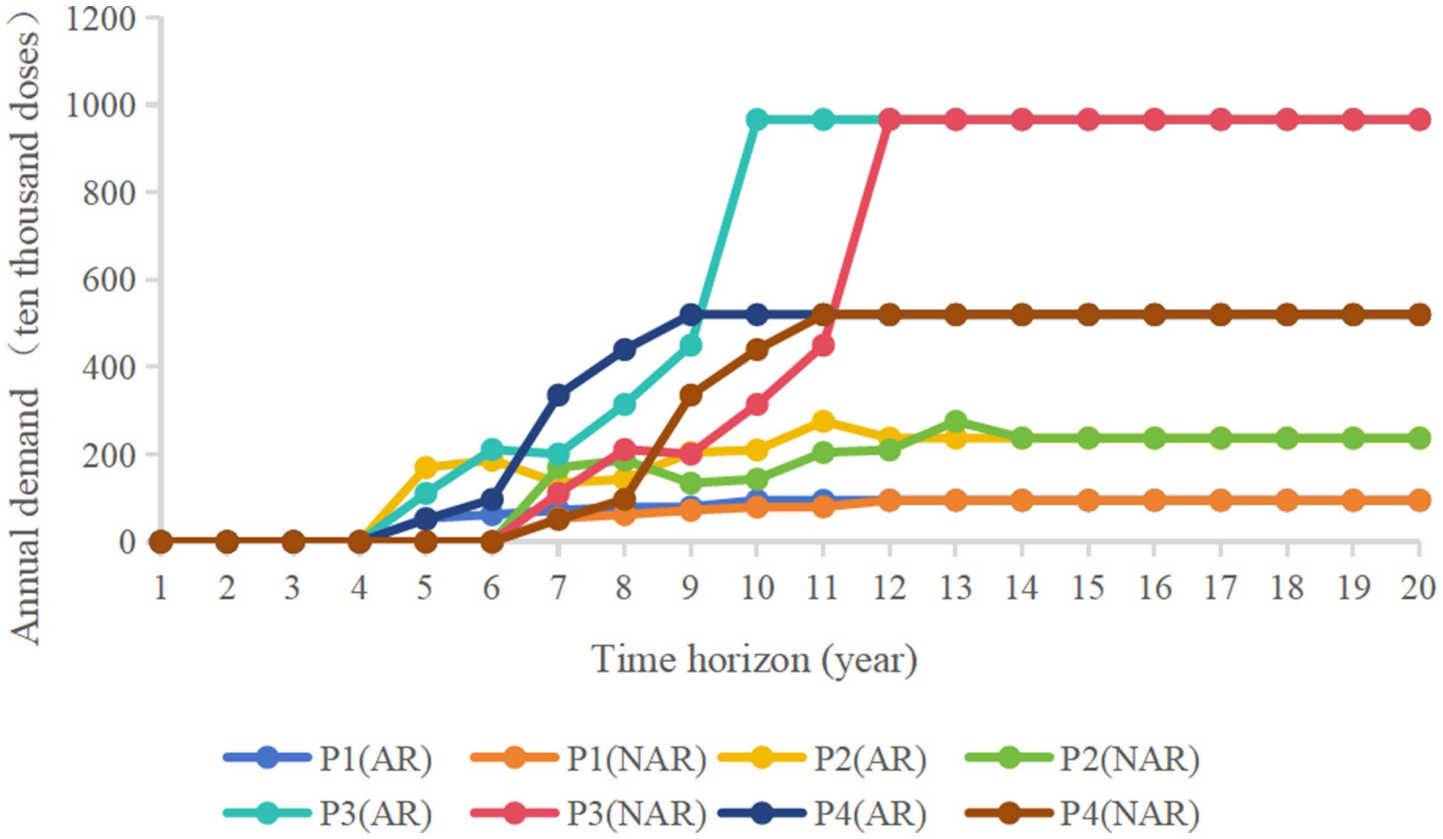
Product demand.

The vaccine products selected for the program are all non-immunization program (Class II) products with no special time period, and the study assumes that if the product is successful in clinical trials, capacity planning decisions will be made in the first year. In order to ensure the scientific soundness of the hypothetical product portfolio, this study conducted consultative interviews with experts and researchers in the pharmaceutical field community, including the pharmaceutical industry, healthcare organizations, government departments, and industry associations, who are authoritative, experienced, and have exposure to, or are very knowledgeable about, vaccines. Table 3 presents a hypothetical product portfolio that lists the product list, demand, unit market selling price, and variable costs. The four product candidates represent the characteristics of currently commercialized vaccine products, with P1 representing a product with relatively low demand and lower sales price. p2 represents a product with a low to medium selling price and medium demand. P3 represents a product with high-value indications and extremely high demand. P4 represents a product with relatively higher value indications and high demand. The idealized annual demand is calculated as a*1/b (a is the total annual demand for a particular vaccine in China in a particular year, and b is the number of companies producing that vaccine), with aP1 = 3, aP2 = 10, aP3 = 1, and aP4 = 10. NAR represents the standard review model, and AR represents the accelerated review policy. Figure 2 shows an annual forecast demand graph based on the annual sales of these four products in the Chinese market in recent years. The graph shows the demand for each of the four vaccine products over a 20-year time horizon as they are approved for marketing through the two policy channels over time. Demand for particular years has been optimized to make the data more representative. Assuming a “rising” demand curve after product approval to model the time it takes for each vaccine product to reach a mature sales level, the annual demand for vaccines levels off and fluctuates up and down within a certain range as education and awareness spreads and sales levels mature.

**Table 3:** Product data.

Product P	Approved indications in China	Annual China demand (ten thousand doses)	Selling Price, Price_P,t_ (RMB)	Cost per dose, VCCOG_P_ (RMB)
P1	Epidemic encephalitis B	94.98	50.00	9.20
P2	Chickenpox	237.39	100.00	10.00
P3	Cervicalcancer (bivalent)	966.67	260.00	21.00
P4	Influenza	520.00	200.00	19.00

#### 6.1.2 Facility equipment data

The research program was designed for two sizes of facilities (36) (Table 4). The small facilities with lower investment costs can only produce P1 and P2, which are relatively simple and less profitable, while the large facilities with higher investment costs can only produce P3 and P4, which are relatively complex but more profitable. According to the constraints, all the facilities can only be put into operation after construction is completed, and other influencing factors such as facility leasing are not considered in this study.

**Table 4:** Facility parameters.

	Small-scale facilities	Big-scale facilities
Annual production (ten thousand doses)	1000.00	3000.00
Investment cost (tens of millions of RMB)	40.70	135.00
Construction period (year)	4.00	7.00

#### 6.1.3 Transport data

In terms of transport costs, vaccine transport is often carried out in two ways: by land in refrigerated trucks and by air in insulated containers, and since this study mainly considers that enterprises directly transport Class II vaccines to other provincial and municipal disease prevention and control institutions, insulated containers are used in the study as the main mode of transport for enterprises by air, and the price is calculated at 70 RMB per kilogram (37), and the average weight of each vaccine is calculated according to the specification of a single dose of vaccine (0.5 mL) + xylitecin vials weight (8 g), totalling 8.5 g. A 50 L insulated box can hold about 770 vaccines, and its weight is about 9 Kg.

#### 6.1.4 Policy time data

Based on the above modeling policy background, it is concluded that the combined use of multiple access modes under the accelerated review policy can shorten the time for clinical development from 7.43 years to 5.62 years and the time for marketing approval from 1.84 years to 1.06 years, relative to the standard review policy. In summary, the accelerated review policy can effectively save enterprises about 2–3 years during the period of promoting new drug development and accelerating the approval of new drugs on the market.

### 6.2 Calculation results and analysis

#### 6.2.1 Comparison of NPV under the two policies

**Figure 3:**
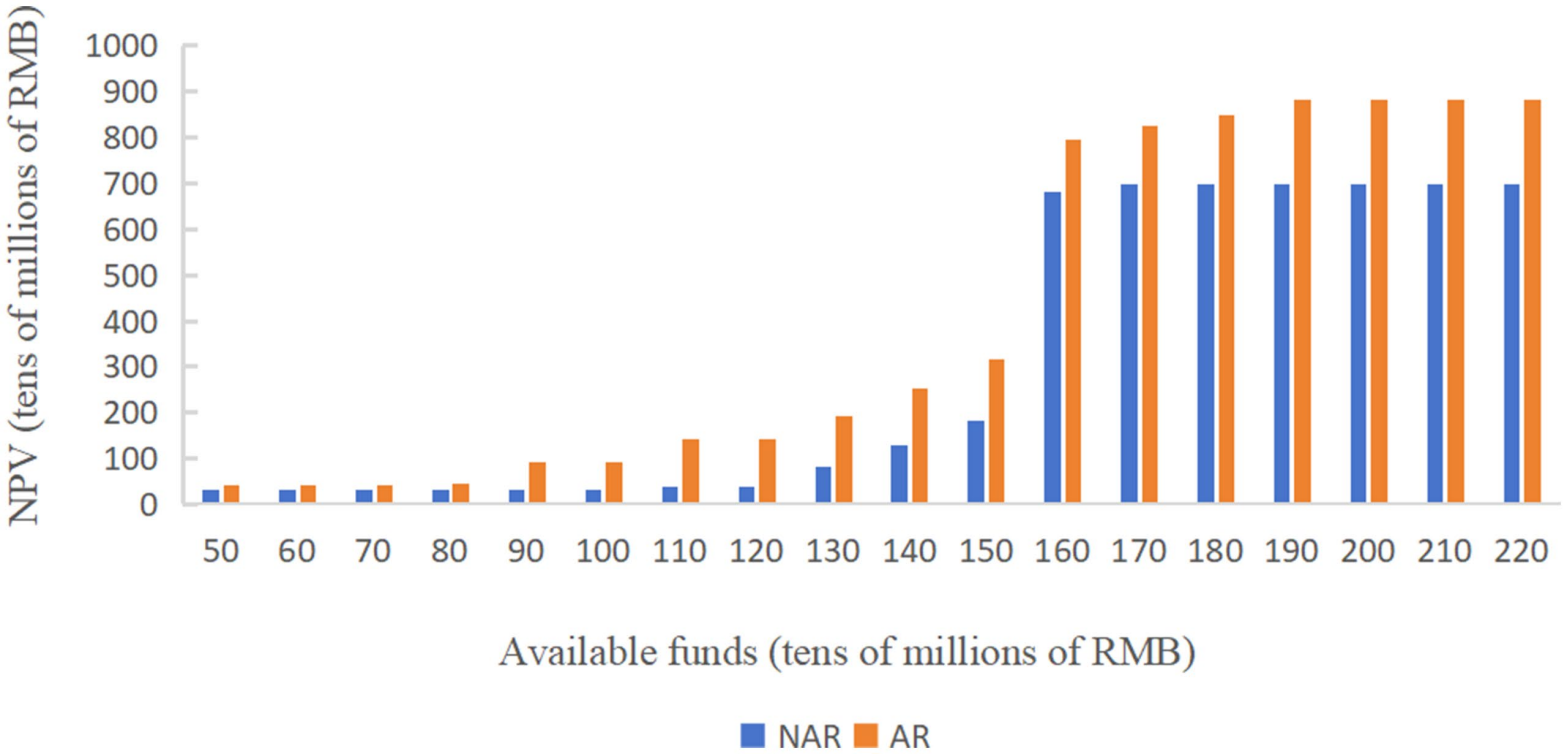
Change in net present value with increase in available funds.

As shown in Figure 3, under the two policy modes, when the available capital is RMB 500–800 million, the NPV in AR mode is slightly higher than that in NAR mode and the difference between the two is less than RMB 100 million, but when the available funds are more than RMB 900 million, the NPV under the AR mode is significantly higher than that under the NAR mode. When the available funds are RMB 1.6 billion, the NPV under both modes shows a cliff-like growth, and after that, the NPV under the NAR mode always tends to level off, while the NPV under the AR mode tends to level off after a small period of continued slow growth.

#### 6.2.2 Optimal investment funds

**Figure 4:**
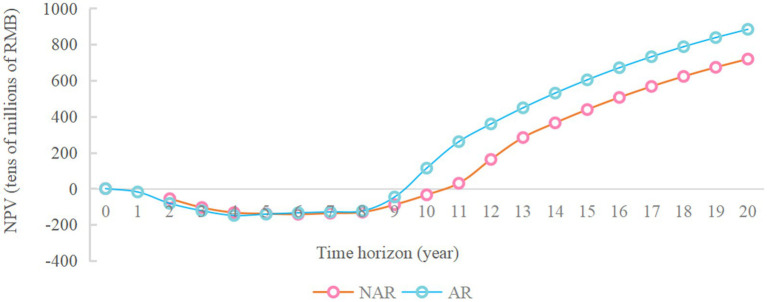
Changes in NPV over a 20-year time horizon limited by RMB 1.9 billion of available funds.

**Figure 5:**
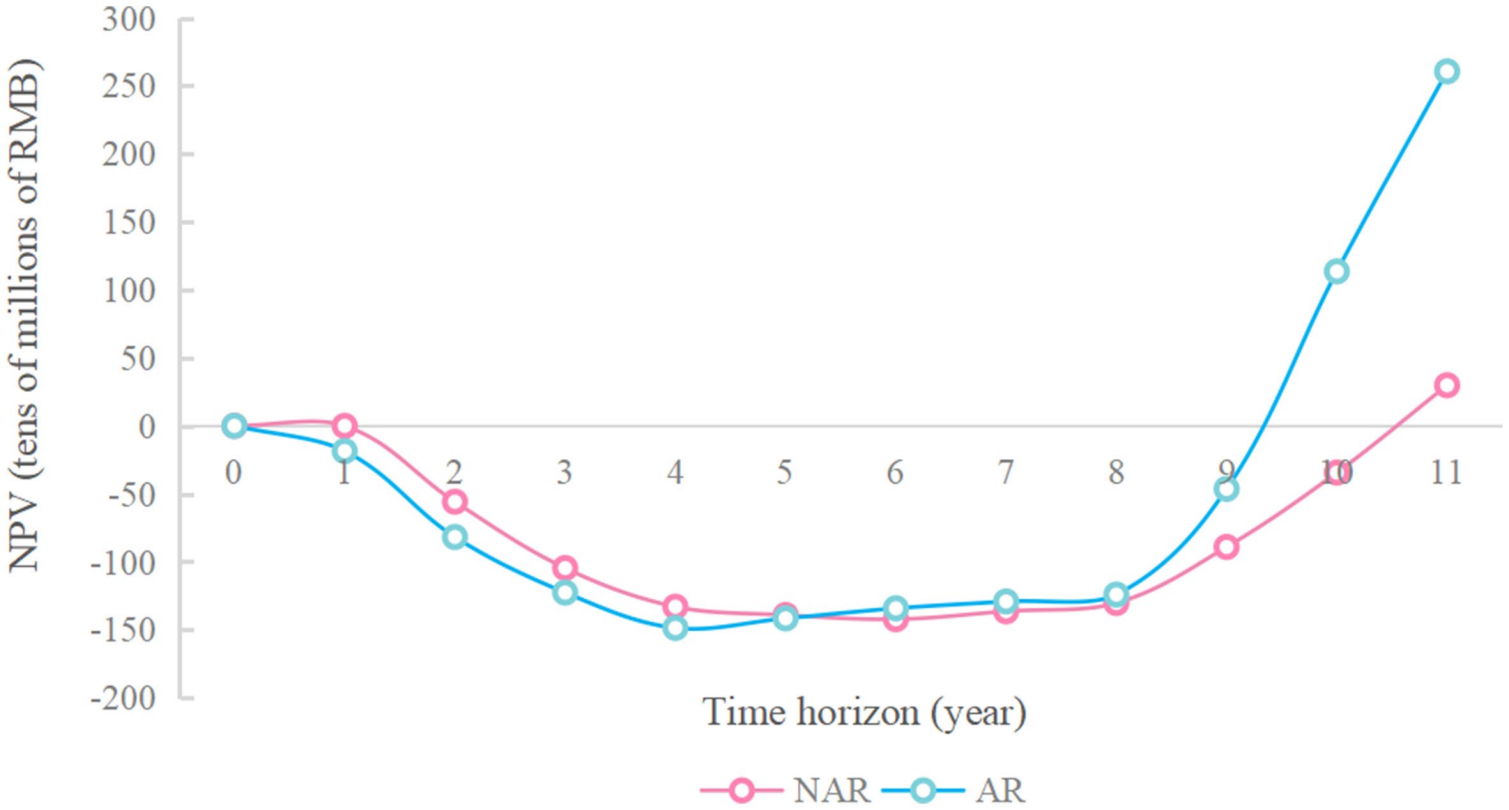
Changes in NPV over the 0-11 year time horizon of the RMB 1.9 billion available funding constraints.

As shown in Figure 3, when the available funds are RMB 1.7 billion, the NPV in NAR mode will reach the maximum value, about RMB 6.97 billion. When the available capital is RMB 1.9 billion, the NPV of the AR mode will reach its maximum value, about 8.84 billion yuan. And the difference between the NPV of the two modes will also reach its maximum value, about RMB 1.87 billion. The NPV of the enterprise during 20 years when the available capital is RMB 1.9 billion is analyzed more deeply. As shown in Figures 4 and 5 (Figure 5 shows an enlargement of Figure 4 for years 0–11), in the early years, the investment under the AR model is stronger and the NPV is smaller, but in the 5th-6th years, the NPV under the AR model starts to reverse; the break-even occurs in about 9.2 years under the AR model, which is about 1.3 years earlier than that under the NAR model, and the NPV value in AR mode is always higher than the NPV value in NAR mode in subsequent times.

#### 6.2.3 Product mix decisions

**Figure 6:**
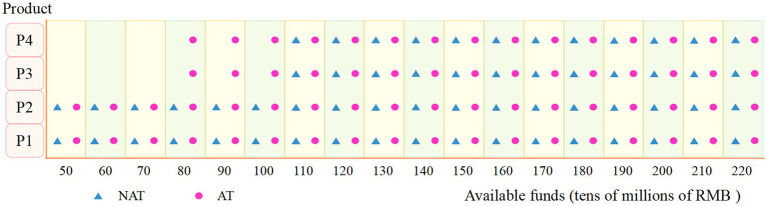
Product portfolio.

Figure 6 illustrates the product mix planning under both models as the available capital increases. When available capital is low, firms will choose to produce P1 and P2, which have lower production costs, simpler processes, and relatively lower selling prices, regardless of the policy model, due to the lower up-front equipment investment costs required for P1 and P2. As available capital increases, firms will begin to choose to produce P3 and P4, which have larger market sizes, more complex processes, and higher selling prices, in order to achieve a higher overall return. However, in the AR model, the firm chooses to produce all four products when the available capital is RMB 800 million, while in the NAR model, the firm chooses to produce all four products when the available capital is RMB 1.1 billion. It can be concluded that with the same available capital, the firm will choose to produce all four vaccines earlier in the AT model to maximize the NPV.

#### 6.2.4 Facility investment time plan

**Figure 7:**
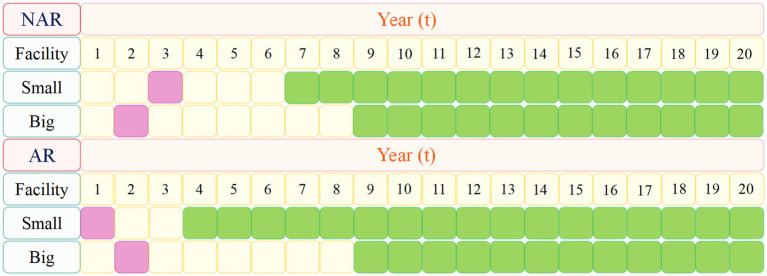
Facility investment time plan.

Figure 7 shows the timing of facility investment plans under the two policy models when RMB 1.9 billion is available. The purple color indicates when the decision to invest in the facility was made and the green color indicates when the facility was available. Firms make the decision to invest in small and large facilities in the AR model in the first and second years, respectively; while the decision to invest in small and large facilities in the NAR model is made in the third and second years, respectively. This is due to the policy of accelerating the market launch of products, enterprises can respond to market demand earlier, so enterprises will make decisions to invest in the construction of facilities earlier under the AR model; while limited by the standard review model of product approval and market launch time is later and the discount factor decreases year by year and other factors, enterprises will make decisions to invest in the construction of facilities later under the NAR model.

#### 6.2.5 Sensitivity analysis

Although the model allows for simultaneous optimization of product selection and market capacity planning, and variations in the input parameters may have an impact on product selection and facility investment decisions, which in turn affects NPV over the entire time horizon, when the available capital is sufficiently large (RMB 1.9 billion was chosen here), variations in the parameters do not affect the firm’s investment decisions on product mix and facilities.

**Figure 8:**
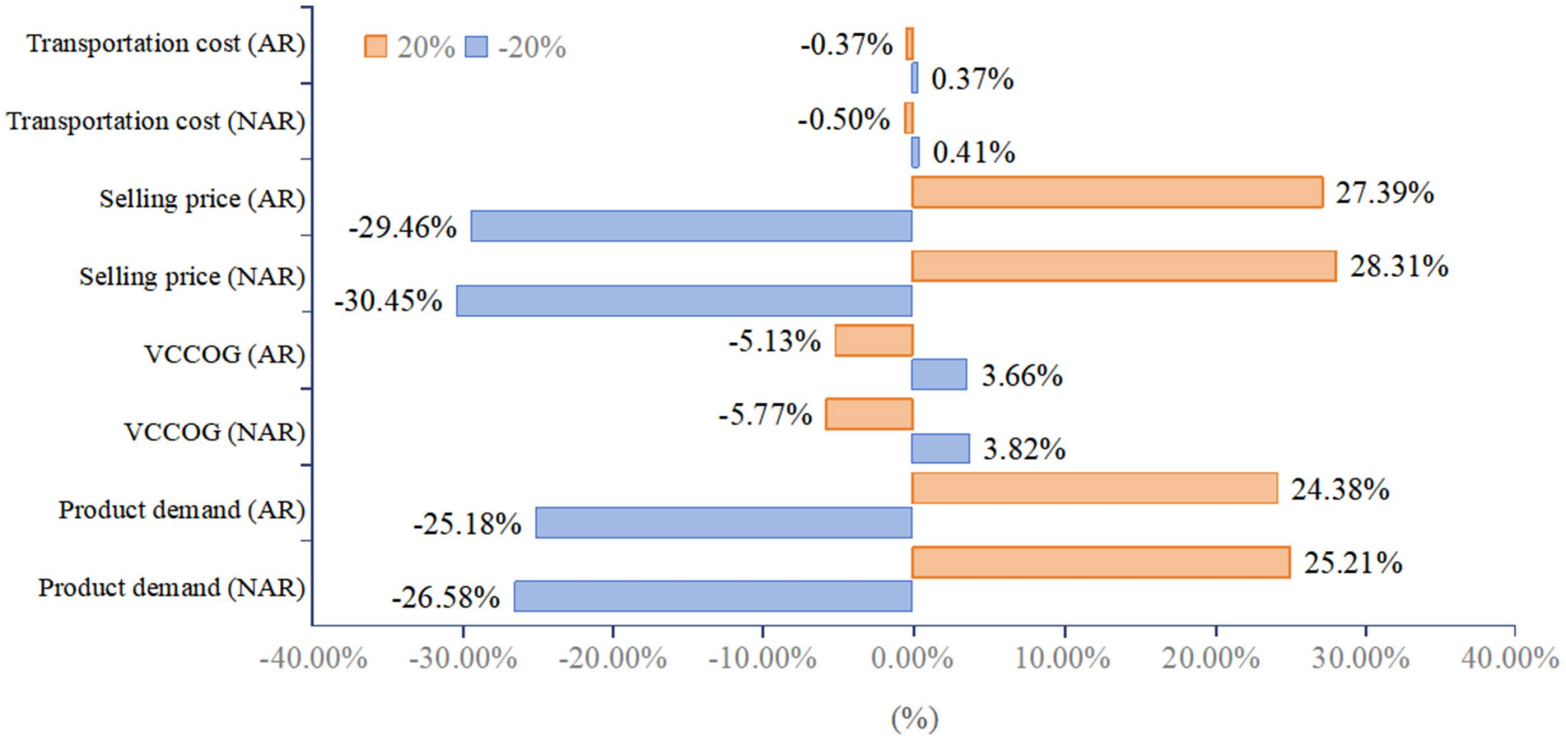
Sensitivity of net present value to parameter changes.

As shown in Figure 8, the overall NPV is most sensitive to selling price, followed by product demand; less sensitive to the total base cost of the product; and least sensitive to transportation costs. The total basic cost of the products selected for the study is generally low, so changes in it do not have as large an impact on NPV as might be expected. Transportation cost is a very small percentage of total cost, so its change has a negligible effect on total cost and NPV. Scenario NAR is more sensitive to sales prices and market demand than Scenario AR (Figure 8).

## 7 Conclusion

In order to investigate the impact of accelerated review policy on the portfolio planning of vaccine enterprises and the differences in the portfolio decisions of vaccine enterprises under the two policy contexts, this paper proposes a new MILP model for portfolio planning of vaccine enterprises and simulates the portfolio decisions of enterprises under different investment funds for a sustained period of 20 years through simulation analysis. The results of numerical experiments show that under the accelerated review policy, although enterprises invest earlier in equipment and products in the early stage, they can achieve breakeven about 1.3 years earlier through the joint use of the four access modes and obtain a relatively higher NPV within the same timeframe. In general, scenes in the accelerated review policy are less sensitive to changes in model parameters. The sensitivity analysis shows a greater than proportionate impact of sales price and product demand on the NPV over the 20-year time horizon for both scenarios.

## 8 Discussion

Considering the recently introduced accelerated review policy and the autologous nature of vaccine formulations, this study proposes a problem-specific solution to the challenges faced by vaccine companies seeking to commercialize vaccine production. We use China as an example to illustrate the impact of the accelerated review policy on capacity planning and investment decisions. Addressing the unique challenges of commercializing vaccine production, the model addresses the timing of investment in facilities of different sizes, long-term capacity investment decisions, and product portfolio decisions. In short, the model can help vaccine companies make rational product portfolio planning decisions in the new context of the accelerated drug review policy introduced in China. Similarly, when constructing derivative models based on the model, enterprises in other countries can also provide reference for their own commercialization strategies to be constructed later by changing the corresponding parameters and variables or adding other conditions according to the similar policy background. At the same time, the results of the model’s operation also provide a reference experience for this country and other countries: enterprises should make full use of the accelerated review policy to gain competitive advantages for themselves in the context of their own R&D products.

## 9 Limitations and future work

One limitation of the modeling study is that it only considers available funds, facility output, product demand, and manufacturing process as constraints, which is clearly not sufficiently considered. Another limitation is that clinical study failures were not considered; in fact, it is unrealistic to assume that every product will be successful in clinical trials. In the future, we may further consider adding new constraints such as location penalty coefficients, refrigerated storage capacity. At the same time, we may continue to expand the model to consider the accelerated review policy contexts in the US, EU, and Japan, and compare the results under different policy environments to provide broader insights into the effectiveness of accelerated review policy globally. In addition, after the outbreak of COVID-19 pandemic, regulators and vaccine companies in various countries are summarizing their experiences and formulating strategies, so in the future, the model can be applied to the CMC strategy of vaccine development, which is widely concerned (36), to help vaccine companies rapidly develop a scientific commercialization strategy, with a view to better preparing for the next pandemic.

## Data Availability

The original contributions presented in the study are included in the article/supplementary material, further inquiries can be directed to the corresponding author.

## Glossary

**Indices**

**Fs -** Facility Scale (Small, Large)

**P -** Products (P1, P2, P3, P4)

**t -** Time periods (years)

**Sets**

**Mkt -** The marketing period after the product is approved (years)

**Parameters**

**Dd_P,t_ -** Total annual demand for a product p in year t, t∈Mkt

**Fixopt_Fs_,_t_ -** Fixed operating cost for a facility with scale Fs in year *t* (RMB)

**Inv_Fs_ -** Investment and construction cost of a facility with scale Fs (RMB)

**M_Fs_ -** Maximum annual production for a facility with scale Fs, which is available after completion

**Price_P,t_ -** Market sales price for product P in year *t* (RMB), t∈Mkt

**VCCOG_P_ -** Total basic cost for a product P, including raw materials, consumables, labor costs, etc. (RMB)

**Year -** Time span of 20 years (t∈Year)

**δ_t_ -** Discount factor in year t

**τf_Fs_ -** Time (in years) needed to prepare for and construct a facility with scale Fs

**Φ -** Available capital (in ten thousand RMB) for the enterprise

**Variables**

**cflow_t_ -** Cash flow in year t (RMB)

**Fac_Fs,t_ -** Quantity of facilities with scale Fs decided to be invested in, in year *t*

**FI_t_ -** Total investment cost for newly constructed facilities in year *t* (RMB)

**NPV -** Net present value of the enterprise within the specified time span (RMB)

**Sales_P,t_ -** Total sales revenue for product P in year *t* (RMB)

**TCOG_t_ -** Total production cost for a product in year *t*

**TFixopt_t_ -** Total fixed operating cost of all available facilities in year t (RMB)

**TRev_t_ -** Total revenue of the enterprise in year *t* (considering only vaccine sales revenue) (RMB)

**TTP_P,t_ -** Total quantity of a product p produced by all available facilities in year *t*

**Binary variables**

**A_Fs,t_ -** Facility availability in year t

**F_Fs,t_ -** Investment decisions for a facility of size Fs in year t
